# 5G Network Slicing: Security Challenges, Attack Vectors, and Mitigation Approaches

**DOI:** 10.3390/s25133940

**Published:** 2025-06-24

**Authors:** José Dias, Pedro Pinto, Ricardo Santos, Silvestre Malta

**Affiliations:** 1 ESTG—Instituto Politécnico de Viana do Castelo, 4900 Viana do Castelo, Portugal; djose@ipvc.pt; 2 ISEP—Instituto Politécnico do Porto, 4249 Porto, Portugal; pfp@isep.ipp.pt; 3 GECAD—Instituto Superior de Engenharia do Porto, R. Dr. António Bernardino de Almeida, 4249 Porto, Portugal; 4 ESTG—Instituto Politécnico do Porto, 4610 Felgueiras, Portugal; rjs@estg.ipp.pt; 5 CIICESI, ESTG—Instituto Politécnico do Porto, 4610 Felgueiras, Portugal; 6 ADiT-LAB—Rua Escola Industrial e Comercial de Nun’Álvares, Nº34, 4900 Viana do Castelo, Portugal

**Keywords:** 5G networks, network slicing, vulnerabilities, security, risk mitigation

## Abstract

This paper explores the security challenges associated with network slicing in 5th Generation (5G) networks, a technology that enables the creation of virtual networks tailored to different use cases. This study contributes to network slicing research efforts by providing a comprehensive classification of attacks aligned with the architectural layers of 5G, complemented by practical mitigation approaches suitable for multi-tenant environments. The classification depicts specific attacks and categorizes vulnerabilities across layers such as orchestration, virtualization, and inter-slice communication. Additionally, mitigation strategies are discussed, emphasizing the importance of real-time monitoring and robust access controls. The proposed classification aims to support the development of advanced security mechanisms, including risk assessment models and automated mitigation strategies, tailored to the dynamic and heterogeneous nature of 5G slicing. The findings highlight the need for layered defenses, AI-driven monitoring, and architectural isolation as critical components to enhance the resilience of 5G slicing deployments.

## 1. Introduction

With the evolution of telecommunications, 5G cellular networks are presented as a revolutionary transformation, promising to impact sectors such as healthcare, transportation, entertainment, and industry [1]. Among its key pillars is network slicing, which enables the creation of virtual networks tailored to different performance and security requirements. However, the complexity of this technology also brings challenges, particularly in terms of security [2]. This paper examines the vulnerabilities of network slicing and proposes mitigation strategies to strengthen its protection in 5G networks.

The adoption of 5G is transforming global connectivity, offering higher speeds, greater capacity, and support for various application scenarios, including enhanced Mobile Broadband (eMBB), Ultra-Reliable Low-Latency Communications (URLLC), and massive Machine-Type Communications (mMTC). Network slicing, one of its core innovations, allows independent virtual networks to operate over shared infrastructure, delivering unprecedented efficiency and flexibility. However, this capability introduces security risks that threaten the expected benefits.

Although network slicing offers significant advantages, its implementation is subject to vulnerabilities that impact Confidentiality, Integrity, and Availability (CIA). Technologies such as Software Defined Networking (SDN) and Network Functions Virtualization (NFV) expand the attack surface, exacerbating the risks. Despite studies on 5G security, there is a lack of specific investigations on network slicing, particularly a detailed classification of attacks, which is essential for developing effective and adaptable solutions. While recent works have begun to address aspects such as authentication mechanisms and lifecycle-based threat models [3,4,5], they do not provide a layered taxonomy of attacks aligned with the architectural structure of slicing or discuss mitigation strategies.

This paper aims to address this gap by

- Exploring the main security challenges associated with network slicing;
- Developing a detailed classification of specific attacks targeting network slicing, identifying threat vectors and associated vulnerabilities;
- Analyzing and proposing mitigation strategies, emphasizing dynamic and real-time adaptive approaches.

This study intends to provide a foundation for future research and enhance network slicing implementations’ resilience.

The paper is structured as follows: Section 2 presents the state of the art, discussing the fundamentals of 5G and network slicing as well as related works and identified gaps. Section 3 explores the main security challenges associated with network slicing. In Section 4, a detailed attack classification is proposed, accompanied by illustrative scenarios and an analysis of mitigation strategies, highlighting current solutions and innovative proposals. Finally, Section 6 synthesizes the study’s contributions and suggests directions for future research.

## 2. State of the Art

The evolution of mobile networks has reached a pivotal moment with 5G, offering groundbreaking advances in connectivity, performance, and versatility [6]. This section explores the fundamentals of 5G technology and introduces the concept of network slicing, emphasizing its importance in facilitating various applications and addressing contemporary connectivity issues.

### 2.1. 5G and Network Slicing

The evolutionary trajectory of mobile networks reflects significant advancements in capabilities and applications [7]. Table 1 summarizes the main generations of mobile networks, their characteristics, and use cases.

Alongside its technological advancements, the 5G ecosystem introduces a wide range of cyber threats stemming from increased virtualization, dynamic resource sharing, and service diversity. Common attack categories include Denial of Service (DoS), unauthorized access, data interception, Application Programming Interface (API) exploitation, and configuration tampering—many of which are amplified in virtualized and multi-tenant environments. Similar threat landscapes have been systematically analyzed in other domains, such as workstation infrastructures, where structured risk assessment methods have proven effective in identifying vulnerabilities and countermeasures [8]. Drawing inspiration from such approaches, this study applies a layered perspective to analyze threats in 5G slicing, with a focus on architectural alignment and practical mitigation strategies.

5G was designed to overcome the limitations of previous mobile generations, offering transformative improvements in speed (up to 10 Gigabits per second (Gbps)), ultra-low latency (under 1 milliseconds (ms)), and support for diverse application scenarios such as enhanced Mobile Broadband eMBB, ultra-reliable low-latency communications URLLC, and massive Machine Type Communications mMTC for IoT. These capabilities enable critical applications across sectors like healthcare, industry, and autonomous systems, while also introducing new security challenges due to the dynamic and virtualized nature of the underlying infrastructure [9].

**Figure 1:**
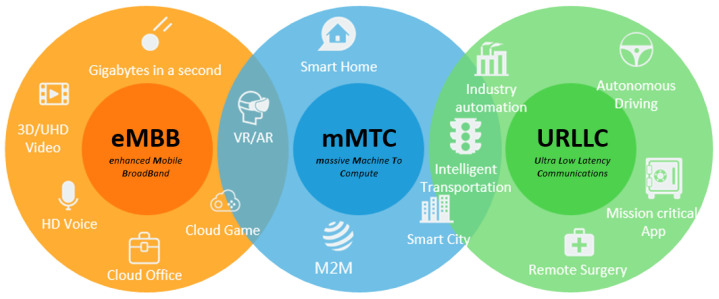
5G—Operational modes. Source: [11].

5G operates in three main modes [10], as illustrated in Figure 1, which provides examples of applications for each mode:

1. eMBB: Applications requiring high bandwidth, such as 8K streaming and virtual reality;
2. URLLC: Essential for time-sensitive and safety-critical applications, including autonomous vehicles, remote surgery, and industrial automation;
3. mMTC: Massive connectivity for IoT devices.

The 5G architecture is built on a service-oriented approach, where various functional entities work together to ensure efficient network operation. These entities are responsible for specific tasks such as authentication, mobility management, data forwarding, and session configuration. The modularity of this architecture enables greater flexibility, scalability, and interoperability among different network components, such as the following:

- User Equipment (UE): Device used by the end-user to access network services;
- (Radio) Access Network ((R)AN): Provides radio access and connects the UE to the core network;
- Access and Mobility Management Function (AMF): Manages user access and mobility;
- Session Management Function (SMF): Configures and controls data sessions;
- User Plane Function (UPF): Forwards and processes data packets in the user plane;
- Unified Data Management (UDM): Manages user subscription and authentication data;
- Authentication Server Function (AUSF): Ensures authentication and security;
- Network Slice Selection Function (NSSF): Selects appropriate network slices for users;
- Network Exposure Function (NEF): Exposes network capabilities securely to external applications;
- Network Repository Function (NRF): Maintains a repository of available network functions and their status;
- Policy Control Function (PCF): Provides policy rules to control network behavior;
- Application Function (AF): Interacts with the core network to support application- level services;
- Data Network (DN): Provides access to external data networks such as the internet or private services.

**Figure 2:**
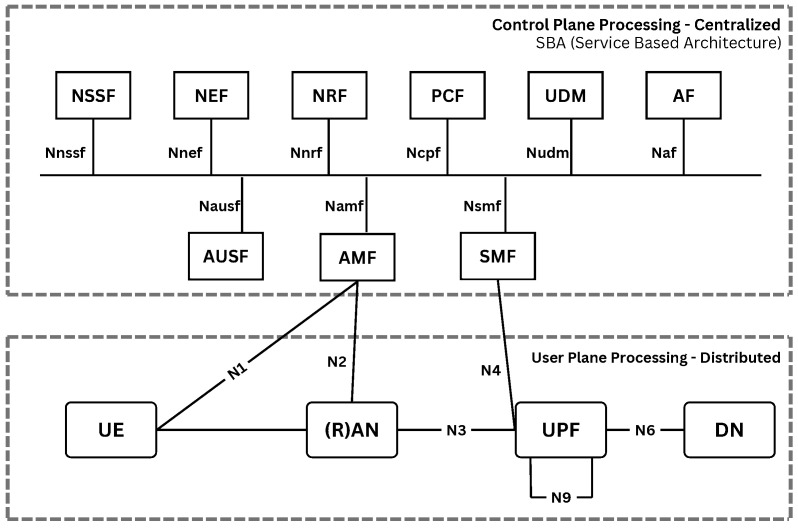
5G—Core architecture. Adapted from [12].

Figure 2 presents the high-level architecture of the 5G core network. It illustrates the logical arrangement of the main functional entities and the separation between the control and user plane, which is fundamental to the service-based architecture of 5G. This structure enables flexible service deployment and efficient resource management.

The operational flow below summarizes how the 5G core network handles registration, authentication, and session establishment for a typical UE. Each step highlights the interaction between the functional entities introduced above:

1. 5G smartphones or mobile devices (UE) connect via 5G NR and subsequently to a DN, such as the internet.
2. The single point of entry for UEs is the AMF, which handles access and mobility procedures.
3. The UE requests a specific service from the AMF, which selects the appropriate SMF to manage the user’s session based on the requested service.
4. The UPF is responsible for routing IP traffic between the UE and the external network (DN).
5. The AMF authenticates the UE by interacting with the AUSF, which validates the user’s identity before allowing access to core services.
6. Additional functions, such as the PCF and AF, enforce network policies and application-level behaviors to ensure proper service delivery [12].

### 2.2. Fundamentals of Network Slicing

Network slicing is a logical segmentation technique that enables the creation of independent virtual networks (slices) over a single physical infrastructure. Each slice is designed to meet specific performance, security, and reliability requirements, as demanded by the different 5G modes [13].

This approach provides network operators with a flexible and efficient way to allocate resources, optimizing infrastructure utilization while ensuring Quality of Service (QoS).

Network slicing operates across three main planes:

- Data Plane (Data plane: Component of the network responsible for forwarding and transporting user data packets.): Routes and processes data traffic between devices and end destinations [14].
- Control Plane (Control plane: Part of the network architecture responsible for signaling, session management, authentication, and policy enforcement.): Manages signaling, mobility, and device authentication [15].
- Management Plane (Management plane: responsible for configuring, monitoring, and orchestrating network resources, including the creation and lifecycle management of network slices.): Oversees the creation, allocation, and orchestration of slices.

The architecture ensures isolation between slices, preventing failures or attacks in one slice from impacting others and providing CIA for each virtual network.

Network slicing is supported by a set of technologies that enable its implementation and large-scale operation. These technologies form the foundation for the flexibility, efficiency, and scalability of this approach, ensuring that the specific requirements of each slice can be met.

- SDN: Separates the control plane from the data plane, enabling centralized and flexible management.
- NFV: Replaces physical network functions with virtual ones, allowing for scalability and dynamic slice creation.
- Orchestration and Automation: Tools such as European Telecommunications Standards Institute (ETSI) Management and Orchestration (MANO) manage slices, allocate resources, and ensure efficiency [16].

These technologies enable the creation of slices and provide the foundation for dynamic orchestration and secure isolation to support the diversity of 5G applications and use cases [17]. The main properties of network slicing are the following:

- Customization and Flexibility: Each slice is configured according to the requirements of the client or application;
- Dynamic Scalability: Slices can be created, adjusted, or deactivated in real time based on demand;
- QoS: Dedicated resources ensure consistent performance levels for each virtual network [18].

### 2.3. Related Work

In [9], the authors explore the key technologies shaping 5G networks, focusing on challenges and opportunities in meeting growing connectivity demands, such as higher capacity, low latency, and IoT integration. It analyzes technologies like massive massive MIMO (M-MIMO), beamforming, device-to-device (D2D) communications, millimeter waves, and small cell densification, highlighting their potential to enhance efficiency and capacity while noting challenges like interference and deployment complexity. The authors address energy efficiency and latency reduction, critical for applications like augmented reality, autonomous vehicles, and machine-to-machine (M2M) communication. They emphasize that transitioning to 5G will require significant changes in base station and network architecture, introducing innovations such as cloud-based radio access networks (C-RAN) and NFV. The study concludes that 5G represents both technological evolution and revolution, demanding advanced solutions to support intelligent applications effectively.

The authors in [19] analyzed recent advances in network slicing, presenting a comprehensive taxonomy, key requirements, and open research challenges. They highlight the role of network slicing in enabling IoT-based intelligent applications in 5G and future networks. The paper covers design principles, resource levels, service function chaining, and security, emphasizing requirements such as elasticity, scalability, and efficient orchestration. It identifies critical challenges, including inter- and intra-slice security, and proposes solutions like federated learning for resource optimization and adaptive business models for slice-based services. The authors conclude that network slicing is crucial for transforming cities into smart, sustainable ecosystems, while calling for new protocols, economic models, and decentralized security mechanisms for Sixth Generation (6G) and beyond.

The authors in [20] explored the use of SDN and NFV in 5G network slicing, discussing key technologies such as Multi-access Edge Computing (MEC) and cloud computing. They identify challenges like slice isolation (slice isolation refers to the ability to ensure that each network slice remains logically and operationally separate from others, preventing unintended interference, data leakage, or unauthorized access), security, and orchestration in multi-domain environments. The paper concludes that network slicing is essential for vertical industries but requires further technical advancements and business model innovation.

The work in [21] introduces a three-dimensional threat taxonomy for NFV in 5G, focusing on the security challenges within an ecosystem involving multiple operators, domains, and complex business collaborations. It identifies critical threat vectors, including vulnerabilities in virtualization, communication links, and service operations. The study also emphasizes the importance of operator collaboration to establish secure 5G ecosystems.

The study in [22] examines security challenges in network slicing for 5G, focusing on threats throughout the slice lifecycle and issues related to intra-slice and inter-slice security. It recommends measures such as slice isolation, robust authentication, and advanced cryptographic techniques to address these risks. However, the authors highlight that significant challenges persist, particularly in ensuring end-to-end security and secure orchestration.

The authors in [23] propose Secure5G, a deep learning framework designed to enhance security in network slicing for 5G and beyond. Their model focuses on proactively detecting malicious connections and mitigating attacks, such as DDoS, before they impact the network. The study highlights the need for dynamic solutions to address the growing complexity and risks of resource sharing among slices, with proposed enhancements targeting security across layers like (R)AN, MEC, and the core.

Authors in [24] by Wajid Rafique et al. examine how Beyond 5G (B5G) networks enhance smart city applications through improved capacity, reduced latency, and support for diverse use cases like transportation, healthcare, and energy management. It provides a taxonomy of the B5G network slicing framework, addressing design requirements, resource allocation, and Artificial Intelligence (AI)/Marchine Learning (ML)-based orchestration techniques. The survey discusses the integration of SDN, NFV, and edge computing for scalable slicing, highlighting challenges such as slice isolation, multi-domain management, and interoperability. Potential solutions include AI-driven automation and distributed management frameworks. This work also reviews real-world implementations and outlines future research directions, emphasizing scalable and adaptive slicing to support smart city demands. This work offers valuable insights for optimizing B5G slicing in urban infrastructure.

The authors in [25] present a Deep Reinforcement Learning (DRL) framework to optimize radio resource allocation in 5G network slicing scenarios characterized by eMBB, URLLC, and mMTC. Their approach adaptively selects between Orthogonal Multiple Access (OMA), Non-Orthogonal Multiple Access (NOMA), and Rate Splitting Multiple Access (RSMA) to meet each service’s distinct QoS requirements under dynamic channel and traffic conditions. By leveraging finite blocklength analysis, the proposed DRL agent learns to allocate frequency resources and determine decoding schemes in real time, maximizing system metrics such as sum rate or successfully decoded devices. Simulation results confirm the effectiveness of this adaptive slicing strategy, showing that it outperforms static approaches in achieving higher network efficiency and reliability.

The work reported in [26] explored how to implement network slicing in Wi-Fi networks to enhance their integration with 5G systems. It proposes a standard-compliant slicing approach using multiple Service Set Identifiers (SSID) per access point to create three distinct slices corresponding to the main 5G service categories: eMBB, mMTC, and URLLC. Two resource allocation algorithms are presented: a static slicing algorithm that allocates fixed resources to slices and a dynamic slicing algorithm that adjusts resources in real time based on network conditions and performance metrics. Extensive simulations using the ns-3 network simulator demonstrate the effectiveness of these algorithms. Results show significant improvements in packet error probability, end-to-end latency, energy efficiency, and spectrum efficiency compared to traditional single-channel Wi-Fi setups. The study concludes that network slicing in Wi-Fi networks, when combined with dynamic resource allocation, can provide better support for diverse application requirements in 5G systems, paving the way for practical implementation in real-world deployments.

The authors in [27] propose a scalable and adaptive framework for network slicing in multi-tenant IoT environments within 5G networks. It addresses the management of heterogeneous slices with diverse QoS requirements, such as latency, reliability, and bandwidth, applied to use cases like smart cities and industrial IoT. Key contributions include a cognitive framework for autonomous slice management, a scalable slicing approach for multi-tenant scenarios, and an enhanced Open vSwitch (OVS) implementation to handle large-scale IoT traffic. The solution demonstrated high scalability in tests, supporting up to 1 million devices, microsecond-level delays for URLLC, over 15 Gbps throughput for eMBB, and average slice creation times of 5.7 ms. The study confirms the framework’s suitability for practical 5G IoT deployments, emphasizing flexibility, scalability, and robust QoS management across diverse applications. Future work will explore real-world scenarios and further performance optimization.

The work presented in [28] examines cybersecurity challenges in 5G network slicing. It highlights how 5G supports IoT applications and critical infrastructures through QoS differentiation, end-to-end security, and big data processing. The study emphasizes the importance of regulations like the Network and Information Security (NIS)2 Directive and international standards while addressing cross-border slicing and the integration of economic incentives and architectural innovations to meet cybersecurity and performance demands in 5G networks.

To summarize the key contributions and limitations of the studies discussed above, Table 2 presents a comparative overview of the related works. It highlights each study’s main focus and contributions, providing a consolidated perspective that supports the identification of existing gaps and the motivation for the approach proposed in this work.

Despite the valuable contributions of these studies, a notable gap remains regarding threats that are slice-specific and that emerge particularly in multi-tenant environments, where isolation and resource sharing introduce new risks. Most existing surveys focus on general 5G security issues or address the enabling technologies (such as SDN and NFV) individually, without providing a comprehensive taxonomy of attacks mapped to the specific architectural layers of network slicing. Moreover, few works have explored how these attacks manifest in operational, dynamic, and heterogeneous slicing scenarios involving multiple tenants. This study addresses this limitation by proposing a structured classification aligned with the functional layers of slicing, highlighting critical vulnerabilities and attack vectors that are unique to this paradigm.

## 3. Security Challenges in Network Slicing

Network slicing in 5G introduces an innovative paradigm that enables logical network segmentation to meet diverse use cases. However, this segmentation also presents significant security challenges. This section explores the most critical challenges, categorized into technical, organizational, and operational aspects.

### 3.1. Technical Challenges

Technical challenges in network slicing arise primarily from the underlying technologies and architectural principles that enable slice creation and management. Issues such as insufficient isolation, virtualization risks, and multi-tenancy complexity can lead to significant security vulnerabilities. This subsection explores the core technical limitations that compromise the confidentiality, integrity, and availability of network slices. Table 3 presents a resume with the security technical challenges in network slicing.

### 3.2. Organizational Challenges

Beyond technical aspects, organizational challenges play a crucial role in the secure deployment and operation of network slicing. These include compliance with standards, integration with legacy infrastructures, and regulatory obligations for data management. Addressing these challenges requires coordinated efforts across operators, vendors, and standardization bodies. Table 4 presents the organizational challenges in compliance and integration in 5G networks.

### 3.3. Operational Challenges

Operational challenges focus on the day-to-day management and resilience of sliced networks. Ensuring real-time threat detection, scalable security, and efficient incident response is essential to maintain service continuity. Table 5 presents the practical limitations that hinder effective security operations in dynamic and heterogeneous 5G environments.

These technical, organizational, and operational challenges highlight the multifaceted nature of securing 5G network slicing. Addressing these issues requires not only technical countermeasures but also structured approaches to identify, assess, and prioritize risks across layers of the architecture.

Security risk assessment plays a critical role in identifying vulnerabilities and prioritizing mitigation efforts, particularly in complex and multi-layered environments such as 5G networks. Recent work by Almuqren [35] demonstrates how structured risk analysis in IoT architectures—through layered threat classification—can effectively uncover attack surfaces and guide countermeasure design. Drawing from this perspective, the present study adopts an architecture-aligned taxonomy to analyze threats in 5G network slicing, aiming to support systematic mitigation and improved resilience.

## 4. Attacks in Network Slicing

The proposed attack taxonomy is structured across four functional layers: Orchestration and Management, Virtualization, Network, and Interface and Communication. This layered approach was chosen to reflect the functional architecture of 5G network slicing, ensuring coverage of the full lifecycle of slice creation, operation, and inter-slice interactions. Each layer captures distinct dimensions of the slicing environment, from resource orchestration to runtime data exchange between slices. By aligning attack vectors with these architectural domains, the taxonomy enables a precise identification of vulnerabilities and facilitates the development of targeted mitigation strategies. In addition, a structured discussion is provided to illustrate real-world attack scenarios, assess their impact, and propose practical mitigation approaches for each vector.

The attack vectors presented in the proposed classification were identified through a structured review of the relevant literature, including academic surveys and technical analyses focused on security threats in 5G network slicing [22], as well as vulnerabilities associated with SDN and NFV technologies [21]. The process involved cross-referencing findings from multiple sources, such as conceptual studies, technical reports, and known software vulnerabilities reported in public databases (e.g., Common vulnerabilities and Exposures (CVE) entries). This approach enabled the identification of recurring risk patterns, including resource contention, insufficient isolation, and insecure API exposure. Although the classification is based on secondary sources, the selected attack vectors aim to reflect threats with practical relevance and alignment to the functional layers of 5G slicing.

This work adopts a qualitative methodology, based on a structured literature review and comparative analysis of secondary sources, to derive and organize attack vectors relevant to 5G network slicing architectures.

**Figure 3:**
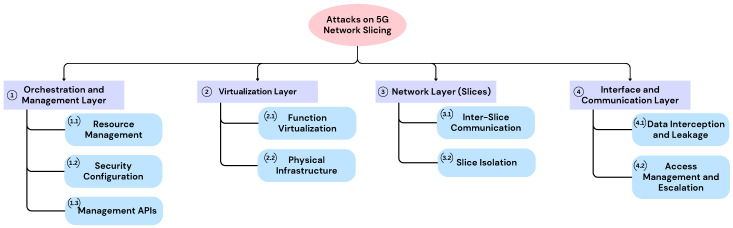
5G slicing attack vector xlassification.

Figure 3 provides a visual representation of the classification, offering a clear and systematic overview of threats to 5G network slicing.

Table 6 complements the proposed classification by detailing specific attack vectors associated with each architectural layer and subcategory. Each entry in the table reflects the numbering system of the classification, ensuring consistency between the hierarchical classification and the identified threat vectors. This structured overview facilitates a clearer understanding of how different attack surfaces relate to the respective network slicing components and highlights critical areas requiring targeted security measures.

## 5. Discussion

This subsection analyzes the most relevant attacks identified in the proposed classification, focusing on those with the highest impact and severity.

Table 7 summarizes key attack scenarios in the context of network slicing, providing real-world examples, practical mitigation strategies, and their mapping to overarching challenge categories. The attacks are structured according to the main attack vectors defined in the classification, offering a clear linkage between vulnerabilities, their exploitation, and suitable countermeasures.

Beyond conceptual risk models, several real-world incidents and proof-of-concept demonstrations have already shown the feasibility and impact of such attacks. A critical vulnerability disclosed by AdaptiveMobile Security [36] showed how flaws in 5G core slicing could allow cross-slice data access and denial of service in multi-tenant deployments. Other studies, such as 5GLatte [37] and 5Greplay [38], demonstrated how poor isolation and weak input validation can enable lateral movement and broad disruptions across both control and data planes.

Attacks targeting the orchestrator, hypervisor (software that enables the creation and management of multiple virtual machines on a single physical host, supporting network function virtualization), and inter-slice communication present critical risks due to their potential to compromise the operation of multiple slices simultaneously. For instance, the orchestrator plays a central role in resource management and allocation, making it a prime target for DoS attacks, resource exhaustion, and configuration manipulation. Similarly, vulnerabilities in hypervisors and APIs can lead to significant exploitation opportunities, especially in multi-tenant environments where isolation between slices may be insufficient.

The exploitation of API vulnerabilities and authentication flaws remains particularly concerning due to their ease of execution and high potential impact, especially when insider threats are involved. These attacks can enable unauthorized access to critical configurations, undermine security policies, and jeopardize the confidentiality and integrity of sensitive data.

Proposed mitigations, such as multi-factor authentication, continuous monitoring, encryption, system hardening, and dynamic resource allocation, are essential to reduce the attack surface and enhance infrastructure resilience. Effective implementation requires a coordinated approach across all layers of network slicing, from orchestration and virtualization to communication and interface management.

Advanced technologies, such as artificial intelligence and anomaly detection, are crucial for enabling proactive monitoring and automated responses to emerging threats. Machine learning (ML) techniques, in particular, can enhance security across the various layers of network slicing. Supervised models may classify benign versus malicious API activity or hypervisor behavior, while unsupervised methods like clustering can detect anomalous inter-slice communication indicative of lateral movement (lateral movement: technique used by attackers to move across systems or slices after initial access, exploiting permissions or segmentation flaws). Additionally, reinforcement learning can dynamically adjust resource allocation or isolation policies in response to evolving attack conditions. Frameworks like Secure5G demonstrate the feasibility of deep learning models in detecting and mitigating attacks within network slicing environments [23]. These AI-driven approaches support early threat detection, contextual decision-making, and responsive mitigation in complex and dynamic 5G infrastructures.

The proposed classification also implicitly spans both the control and data planes, even if not explicitly segmented that way. Categories such as resource management, management API, and security configuration typically reflect control plane threats related to orchestration and policy enforcement. In contrast, vectors like inter-slice communication, interception and data leakage, and physical infrastructure target the data plane, impacting the actual flow and confidentiality of user traffic. Some categories, such as function virtualization or slice isolation, may span both planes depending on the specific attack. This cross-plane and cross-layer perspective enhances the taxonomy’s relevance for comprehensive risk assessment and defense design in complex 5G environments.

In summary, the analysis underscores the importance of prioritizing attacks with the greatest severity and impact, particularly those targeting critical layers such as orchestration, resource management, and inter-slice communication. A combination of robust preventive measures and dynamic mitigation strategies is key to ensuring the security and operational continuity of 5G networks.

## 6. Conclusions and Future Perspectives

This work presents a comprehensive analysis of the security challenges inherent to network slicing in 5G networks and proposes an attack classification with the vulnerabilities that compromise the confidentiality, integrity, and availability of this technology. The exploration of orchestration, virtualization, and inter-slice communication layers reveals the complex dependencies that exacerbate security risks in multi-tenant environments.

The proposed classification provides a structured approach for understanding and categorizing potential threats, offering researchers and practitioners a foundation to address these challenges methodically. Furthermore, the analysis of mitigation strategies emphasizes the importance of real-time monitoring, robust access controls, and dynamic resource allocation as key measures to strengthen network slice security.

Despite these contributions, several challenges remain unresolved. Ensuring end-to-end security, achieving seamless integration with legacy networks, and addressing the evolving threat landscape in the context of 6G will require ongoing research and innovation. The development of AI-based tools for threat detection and automated responses will play a crucial role in securing future network slicing implementations.

This work intends to contribute to the growing body of knowledge on network slicing security, providing a foundation for future advancements and promoting the resilience of next-generation communication networks.

## Figures and Tables

**Table 1:** Evolution of mobile technologies.

Gen.	Launch Year	Main Technology	Characteristics	Use Cases
1G	1980s	Analog Voice	Low-quality voice communication	Simple voice calls
2G	1990s	GSM	Digital voice, SMS, and improved security	Text messages and calls
3G	2000s	UMTS / WCDMA	Mobile data, introduction of mobile internet	Web browsing, basic multimedia
4G	2010s	LTE	High-speed data, support for HD video streaming	Streaming, video conferencing, apps
5G	2020s	NR / Virtualized Core Networks	Low latency, high bandwidth, massive IoT, URLLC	IoT, autonomous vehicles, AR/VR, Industry 4.0
6G	2030s	Terahertz (THz) Networks, AI-Integrated Networking	Sub-millisecond latency, holographic communication, speeds > 1 Tbps	XR, distributed neural networks, smart cities

**Table 2:** Comparison of related works.

Article	Main Focus	Contributions
[9]	Emerging technologies for 5G networks	Analysis of key technologies, challenges, and opportunities for 5G, such as M-MIMO, D2D, and millimeter waves.
[19]	Taxonomy, requirements, and challenges of network slicing	Comprehensive taxonomy of the network slicing framework and future directions.
[20]	Use of SDN and NFV in 5G network slicing	Comprehensive review of enabling technologies, use cases, and future challenges.
[21]	Security in NFV-based 5G networks	Three-dimensional threat taxonomy and analysis of business model and security gaps.
[22]	Security in network slicing in 5G networks	Analysis of threats throughout the lifecycle, including intra-slice and inter-slice, and proposed mitigation recommendations.
[23]	Security in network slicing using deep learning	Secure5G model to detect and mitigate attacks, including DDoS, and dynamic isolation of malicious traffic.
[24]	Beyond 5G network slicing for smart cities	Taxonomy of B5G slicing, design requirements, AI/ML-based orchestration, and resource allocation for smart cities.
[25]	Resource allocation and decoding scheme selection in 5G slicing	Proposes a DRL-based approach to dynamically choose among OMA, NOMA, and RSMA for eMBB, URLLC, and mMTC traffic.
[26]	5G network slicing for Wi-Fi networks	Proposal of a slicing approach using SSID in Wi-Fi networks, with static and dynamic resource allocation algorithms.
[27]	Adaptive network slicing in multi-tenant 5G IoT networks	Scalable framework for heterogeneous slice management, cognitive slice management, and Open vSwitch-based implementation.
[28]	Governance of cybersecurity in 5G network slicing	Analysis of economic and regulatory challenges, the NIS2 Directive, and cross-border slicing security governance.

**Table 3:** Technical challenges: Security issues in 5G network slicing.

Issue	Description	Impact	Cause
Slice Isolation Issues	Slice isolation is essential to prevent failures or malicious activities in one slice from impacting others. However, shared resource utilization makes full isolation challenging.	Data leakage or cross-slice attacks (e.g., side-channel attacks) may compromise security and privacy.	Inadequate use of SDN and NFV, which were not designed for granular segmentation [29].
SDN and NFV Vulnerabilities	SDN and NFV, core technologies for slicing, introduce specific vulnerabilities such as centralized SDN controllers and weak VNF configurations.	These vulnerabilities expose the infrastructure to DoS attacks and increase the attack surface.	Result from implementation complexity and lack of robust validation [30].
Multi-Tenancy Complexity	Multi-tenancy allows multiple providers to share the same infrastructure in isolation but increases security risks.	Malicious tenants can exploit vulnerabilities to compromise others or interfere with security policies.	Poorly configured or inadequate security policies in shared environments [31].
Virtualization and Shared Resources	Virtualization increases the potential for side-channel attacks and resource overloading.	Poor resource allocation can compromise QoS across multiple slices.	Lack of granular control over shared resource usage [32].

**Table 4:** Organizational challenges: Compliance and integration challenges in 5G network slicing.

Issue	Description	Impact	Cause
3GPP Standards and Compatibility	Adhering to 3GPP standards is essential for interoperability and security.	Implementation discrepancies can lead to security gaps.	Coexistence with legacy technologies and the complexity of emerging standards [33].
Integration with Existing Networks	Legacy 4G networks connected to 5G can expose old interfaces to new threats.	Vulnerabilities in legacy networks can be exploited to compromise 5G slices.	Differences in security approaches between network generations.
Data Management and Compliance	Managing sensitive data in sliced networks must comply with regulations such as GDPR.	Violations can lead to legal penalties and loss of user trust.	Lack of tools for continuous auditing and monitoring [34].

**Table 5:** Operational challenges in 5G network slicing.

Issue	Description	Impact	Cause
Real-Time Monitoring and Management	Continuous monitoring is essential to identify and mitigate threats.	Lack of effective solutions allows undetected attacks to affect multiple slices.	Absence of integrated tools for real-time monitoring.
Incident Response and Recovery	Interdependencies between slices hinder rapid and efficient incident response.	Failures can disrupt critical services like URLLC.	Lack of standardized protocols for incident response.
Scalability and Flexibility in Security	Sliced networks must dynamically adapt to traffic and QoS changes.	Static solutions may fail to meet growing demands.	Limited use of technologies like AI for adaptive security.

**Table 6:** Security threats across 5G network slicing architecture layers.

Layer	Subcategory	Description of Threat
1—Orchestration and Management Layer	**1.1—Resource Management**	Attacks aim to disrupt resource allocation across slices, often through resource exhaustion (e.g., flooding orchestrators with fake requests), leading to service degradation or DoS. Attacks on orchestrators or managers can result in unauthorized reallocation or slice disruption.
1—Orchestration and Management Layer	**1.2—Security Configuration**	Exploiting vulnerabilities in orchestrators (e.g., OSM) can lead to unauthorized access and misconfigured slices. Attackers may weaken security policies such as authentication and encryption to escalate privileges or extract data.
1—Orchestration and Management Layer	**1.3—Management APIs**	Poorly secured APIs enable attackers to access slice management functions. Weak authentication or excessive permissions can lead to unauthorized modifications, data leakage, or service disruption. Real incidents have shown how exposed APIs in orchestration platforms pose significant risks.
2—Virtualization Layer	**2.1—Function Virtualization**	Attacks on the hypervisor or NFVs can allow privilege escalation, manipulation of services, or code injection. This may result in instability or outages across multiple slices sharing infrastructure.
2—Virtualization Layer	**2.2—Physical Infrastructure**	Hardware-level attacks (e.g., tampering or DoS on components) can disrupt all slices dependent on the affected infrastructure due to the shared nature of physical resources.
3—Network Layer	**3.1—Inter-Slice Communication**	Poor isolation may lead to unintended data exchange or unauthorized access between slices. Attackers can exploit these flaws to intercept traffic, leak data, or breach isolated services.
3—Network Layer	**3.2—Slice Isolation**	Resource-sharing mechanisms may be abused to exhaust shared resources, degrading performance, or allowing lateral movement across slices. Weak dependency controls increase this risk.
4—Interface and Communication Layer	**4.1—Interception and Data Leakage**	Techniques such as traffic analysis or credential theft can be used to monitor operations or extract sensitive data. Internal threats and weak security controls contribute to these risks.
4—Interface and Communication Layer	**4.2—Access and Escalation Management**	Weak authentication or command injection can lead to unauthorized access or privilege escalation. Attackers may move laterally across slices, gaining deeper control over the network.

**Table 7:** Relevant attacks, examples, and mitigations.

Subcategory (Service Affected)	Challenge Category	Practical Example	Mitigations
Resource Management	Technical	Flooding with false requests leads to orchestrator unavailability.	Traffic monitoring and rate-limiting mechanisms.
Security Configuration	Technical	Modification of slice configuration policies to weaken security.	Strict access controls and validation of configuration changes.
Management APIs	Technical	Exploitation of misconfigured APIs to gain unauthorized access.	Strong authentication, permission checks, and API hardening.
Function Virtualization	Technical	Exploitation of hypervisor vulnerabilities to control virtual machines.	Hypervisor hardening and implementation of segmentation techniques.
Physical Infrastructure	Technical	Direct attacks on physical components causing service disruptions.	Regular maintenance and physical security measures.
Inter-Slice Communication	Technical	Data interception between slices due to the lack of encryption.	Encryption protocols for data in transit and secure communication.
Slice Isolation	Technical	Resource exhaustion in one slice affecting multiple slices.	Dynamic resource allocation and slice isolation mechanisms.
Data Interception and Leakage	Operational	Traffic monitoring to infer usage patterns and obtain private data.	Traffic encryption and anomaly detection systems.
Access Management and Escalation	Operational	Exploitation of authentication mechanisms to access critical slices.	Multi-factor authentication and real-time access monitoring.

## Data Availability

The original contributions presented in this study are included in the article. Further inquiries can be directed to the corresponding author.
